# Humanizing the Transgressor and Lightening the Immoral Behavior: The Role of Likeability Bias and Moral Rationalization

**DOI:** 10.3390/bs14121206

**Published:** 2024-12-16

**Authors:** Sofía Moreno-Gata, Ramón Rodríguez-Torres, Verónica Betancor, Armando Rodríguez-Pérez

**Affiliations:** Department of Cognitive, Social and Organizational Psychology, Faculty of Psychology, University of La Laguna, 38200 San Cristóbal de La Laguna, Spain

**Keywords:** likeability bias, moral rationalization, dehumanization, severity of the behavior, immorality, bystander

## Abstract

People often perceive their moral judgments as objective and unbiased, yet research indicates that positive interpersonal attitudes lead to more lenient moral character assessments. Here we investigate how likeability towards moral transgressors and the different moral rationalization strategies they may employ impact both the perceived severity of the immoral behavior and the attribution of humanity to the transgressor. In two studies, participants (*N* = 475) engaged in a 2 (likeability towards the transgressor: high vs. low) × 2 (moral rationalization: reconstruction of agency vs. reconstruction of morality) between-subjects experiment. Participants read information about an individual and an immoral action they engaged in and then evaluated the severity of the behavior and the degree of dehumanization of the transgressor. Results showed that feelings of likeability towards the transgressor, as well as rationalizing by reconstructing agency (compared to morality) reduced behavior severity and transgressor dehumanization. Moreover, likeability and the use of agency reconstruction by the transgressor showed an additive effect, as they combined to generate the most benevolent judgments. Recognizing the influence of these variables enhances our understanding of moral decision-making processes in interpersonal contexts.

## 1. Introduction

Social life is regulated by normative systems that guide interactions and establish the necessary mechanisms to correct any transgressions that may occur. Among these systems, moral norms play a fundamental role. These norms allow us to distinguish between good and bad actions and prevent harm to others, the community, and its symbols or institutions [1]. In fact, a behavior that violates the common sense of what is right and wrong is judged as a moral transgression and a threat to social cohesion and the proper functioning of the community. For this reason, in recent decades, social psychologists have begun to investigate some of the processes underlying moral decision-making.

Thus, today, we have a vast knowledge of the relationship between moral judgments and emotions [1,2], personality [3], religious beliefs [4], cultural norms [5,6,7], and socialization [8]. Research also studies the relationship between moral judgments and attitudes. However, while much of this work has mainly focused on examining how morality influences attitudes (e.g., [9,10,11,12]), many questions remain about how attitudes influence moral judgments [13,14]. In this study, we aim to further contribute to this topic by examining how people’s attitudes towards a transgressor influence the outcomes of the moral judgement following a transgression. Furthermore, in analyzing moral judgement, we go beyond severity of the misbehavior to also study dehumanization of the transgressor.

For a long time, people have thought that moral norms are universal convictions, objectively true and immune to preferences and attitudes. Consequently, they have perceived their moral judgments as objective decisions, akin to scientific facts [15,16]. However, recently, some authors have been working in an innovative direction regarding the relationship between attitudes and moral judgments. In this line of research, Bocian et al. [17] indeed found evidence of the influence of interpersonal attitudes on moral judgments. Specifically, through four studies, they showed that mere liking of a person elicited a more lenient moral judgement of that person. That is, participants tended to consider individuals they liked as having better moral character compared to those they did not like, even though both engaged in the same behavior. These data suggest that interpersonal attitudes strongly influence judgments about the behavior and moral character of others, even though people believe in the objectivity of their moral beliefs. Moreover, the effect of mere liking is important because it is a prevalent and immediate dimension in first impressions [18] and is strongly connected to the moral information conveyed about people [19,20].

Considering these precedents, the present research is interested in studying the effect that a favorable attitude towards the transgressor has on two reactions involved in the moral judgement process. On the one hand, we analyze the degree of severity attributed to the transgression. On the other hand, we examine the perception of the transgressor themselves, more specifically, to what extent they are dehumanized.

Regarding the evaluation of severity, studies show that an action is judged as more or less severe depending on the circumstances and perspectives of the observers [21,22,23,24]. Specifically, researchers in this field have observed that some positivity biases cause us to perceive the actions of people we feel affection towards as less severe and interpret their actions with more leniency. Perceptual biases have also been observed that minimize the intentionality and malice of the transgressor by attributing the cause of their behaviors to situational factors [25,26,27].

However, liking the transgressor not only makes bad behaviors seem more benign, it also increases positive expectations about the moral character of the transgressor [28]. Does this mean, then, that a “likeable” moral transgressor could restrain observers’ tendency to dehumanize them? Studies on animalistic dehumanization [29] establish that morality, along with civility, refinement, rationality, and maturity, is an exclusive and necessary characteristic to be considered a human being. Consequently, the lack of morality or the commission of immoral acts is sufficient to place a person or group on a lower rung, in a subhuman category [30,31,32,33]. Moreover, the way people perceive others as more or less human is surprisingly flexible [30] and often occurs implicitly. Thus, people tend to attribute complex secondary emotions (e.g., hope or guilty), seen as uniquely humans, more to in-group members, while associating basic primary emotions (e.g., joy or fear), which humans share with other animals, equally with both in-groups and out-groups [32]. In fact, this is what both victims and observers of immoral actions do. The former dehumanize their aggressors as a mechanism to reduce their intentionality and status and increase social distance from them [34,35]. The latter do so to short-circuit empathy towards them [36,37] and to have more reasons to propose longer and harsher sentences and less confidence in their rehabilitation [38,39].

Surprisingly, to date, no research has tested whether this tendency to dehumanize those who engage in immoral behaviors collapses when there are positive attitudes towards the transgressor. Additionally, what would happen if the transgressor presented themselves as someone capable of rationalizing the bad action they have performed? So far, research interested in the moral evaluation of behaviors (e.g., [40]) presents transgressions regardless of the reasons the transgressor gives for their action, as if it were possible to avoid the strong rationalizing pressure people experience after engaging in immoral behavior [41,42]. However, usually, after inappropriate behavior, people create “useful fictions” designed to neutralize the negative value of their actions and maintain an image of themselves as moral and rational individuals in their own eyes and in the eyes of others [41,42]. These post-hoc explanations are effortful processes aimed at justifying the action, even when a moral judgement, whether intuitive or deliberate, has already been received [1]. Consequently, people tend to employ different strategies to disconnect their moral standards from their behaviors [43], alleviate their feelings of guilt, and justify their questionable actions [44,45]. According to the moral disengagement perspective, this process operates dynamically through eight distinct mechanisms across four loci of the self-regulatory system: conduct, agency, consequences, victim. In an effort to enhance parsimony, Schaefer and Bouwmeester [46] categorized these mechanisms into two dimensions, suggesting that these fictions can seek to neutralize the transgressor’s malice by arguing that the agent is not responsible for the behavior (agency reconstruction) or to neutralize the malice of the action by arguing that the behavior is not morally wrong (morality reconstruction). Investigating whether both types of rationalization strategies produce similar results is important, as previous studies indicate that strategies absolving the agent of responsibility are more effective in repairing trust than those framing the behavior as not so wrong [47]. Moreover, the Theory of Dyadic Morality states that agency (alongside harm and vulnerability of the victim) is one of the three main components of judging an action as immoral [48]. Accordingly, we expect that rationalizing by saying that the transgressor had no responsibility will make the act seem less severe and the transgressor appear more human, compared to presenting the behavior as less wrong.

Thus, in this research, we aim to contribute to the existing knowledge in this field by examining the effect of liking a transgressor who rationalizes their action on both the perceived severity and the attribution of humanity to that transgressor. Specifically, we are interested in assessing the effect of positive and negative attitudes on the evaluation of immoral transgressions when these are followed by different types of moral justification.

### Overview of the Present Studies

Although previous research has emphasized the importance of liking in behavior evaluation, as far as we know, the effect of liking on the perceived severity of an action and the attribution of humanness to individuals who commit immoral acts has not been studied. Furthermore, most studies on moral transgressions present actions independently of the justifications that transgressors use to maintain both their moral identity and social ties. In two studies, we propose that the likeability of the transgressor and the moral rationalization they employ bias the perceived severity of immoral behavior and the tendency to dehumanize the immoral agent.

Specifically, regarding the severity of the behavior, we argue that as the transgressors are perceived as more likeable, the perceived severity of their actions is lower (H1). Moreover, using reconstruction of agency rather than reconstruction of morality is also associated with decreased perceived severity of the transgression (H2). Finally, we anticipate an interactive additive effect of both factors, such that the actions of those more likeable transgressors who use the reconstruction of agency are judged the least severely (H3). Our hypotheses concerning dehumanization follow a similar line. Specifically, we argue that the more likeable the transgressors are perceived to be, the less they will be dehumanized (H4). Furthermore, using reconstruction of agency rather than reconstruction of morality is also associated with decreased dehumanization of the transgressor (H5). Finally, we expect an interactive additive effect of both factors, such that the more likeable transgressors who use the reconstruction of agency will be dehumanized the least (H6).

To test the hypotheses, we conducted two studies manipulating likeability using two different procedures. Study 1 focused on personal likeability by presenting participants with a transgressor with either a positive or negative personal profile, using the three dimensions of the extended stereotype content model. In Study 2, likeability was manipulated by presenting participants with a transgressor who either shared or did not share their ideological preferences. In both studies, the transgressor rationalized their behavior by reconstructing either the agency or the morality of the action.

## 2. Study 1

The objective of Study 1 was to determine whether the positive (or negative) profile of a moral transgressor can bias the perceived severity of the immoral action and the attribution of humanness to the transgressor. To generate a positive impression of the transgressor, this study was based on research demonstrating that everyday social judgments are constructed around three fundamental evaluative dimensions. Specifically, Brambilla et al. [49] found that information about competence, sociability, and morality was capable of generating an overall positive evaluation of the described person (see also [50]). Of particular interest was the finding that information about morality had a more potent impact on this overall evaluation, as it eliminated the perception of threat that information about competence might pose [19]. In addition, using fictitious groups where traits of competence, sociability, and morality were manipulated, a significant positive correlation was found between the scores given to individuals in these three dimensions and the overall evaluation of them: the more competent, sociable, and moral the individuals were perceived to be, the better they were evaluated [20]. Consequently, to facilitate the process by which an organized and positive general impression emerges from individual traits, this study provided participants with a description of a person with a positive (or negative) profile in the traits listed in the three dimensions of the extended stereotype content model [19].

### 2.1. Methods

#### 2.1.1. Participants

British participants were recruited through an online research recruitment platform in exchange for a financial reward, and Qualtrics hosted the survey online. According to the G*Power analyses for the F tests (ANOVA: fixed effects, special, main effects, and interactions) with *p*-values α < 0.05, 4 groups, numerator *df* = 1, 95% power, and a medium effect size (*f* = 0.25), the target sample size should be 210 participants. Assuming an error in the manipulation and attention check responses, we aimed to recruit a total of 280 individuals. Then, data were screened to exclude participants who did not recognize the type of moral reasoning employed to justify the immoral action, failed the attention check questions, or stated themselves not to analyze their answers. Therefore, the final sample consisted of 229 participants with a mean age of 41.57 (*SD* = 13.80) and a gender breakdown of 49.80 % women, 49.80 % men, and 0.40 % other.

#### 2.1.2. Design

To test our hypotheses, we conducted a 2 (profile of the target: positive vs. negative) × 2 (moral rationalization: reconstruction of agency vs. reconstruction of morality) between-subjects experiment on the severity of the behavior and dehumanization of the transgressor variables.

#### 2.1.3. Material

##### Stimuli

(1) Likeability of the Target

The degree of likeability of the target was manipulated using two brief descriptions of an individual. Participants were informed that in a prior study, we conducted focus group discussions wherein diverse participant groups described an acquaintance they had in common and knew well. We pretended that we collected information about various individuals from these discussions and presented participants with a single profile. To construct the profile, two brief passages of 113 words each were written. Adjectives from the three dimensions of the extended Stereotype Content Model were used (morality: sincere, honest, trustworthy; sociability: friendly, warm, likeable; competence: intelligent, competent, skilled) [19,50]. The positive profile highlighted favorable traits of the target, named “LM”. For instance, regarding moral traits, the following was shared: “From the start, they said LM’s sincerity and honesty shine through. LM projects a level of trustworthiness that is both comforting and reliable, making them someone you can unquestionably rely on” (see full materials at OFS). For the negative profile, the opposite traits were used (e.g., dishonest, incompetent, etc.), and we adjusted the narrative accordingly: “From the start, they said LM’s insincerity and dishonesty shine through. LM projects a level of untrustworthiness that is both unsettling and unreliable, making LM someone you can hardly rely on”.

(2) Behaviors

The moral transgressions were selected from a normative study [51], where participants evaluated moral transgressions on severity and inappropriateness, among other variables. Based on that data, we chose two transgressions that were judged as equally severe (*t* (42) = 1.15; *p* = 0.256) and inappropriate (*t* (42) = −0.77; *p* = 0.445): “Starting a false rumor about someone” and “Having sex with a friend’s significant other”.

(3) Moral Rationalization

Each of the selected behaviors was followed by an explanation attempting to justify the transgression, either by absolving the agent or neutralizing the immorality of the action. For instance, the participants who were informed that an agent had started a false rumor about someone also read that the agent said the following: “Actually, I didn’t start the rumor. It was someone else, but they claimed it was me” (an argument that absolves the agent of responsibility) or “I don’t think it matters. People say things about everyone that aren’t true” (an argument that diminishes the immorality of the action).

To confirm that each argument accurately represented the distinction between reconstructing the agent’s responsibility or reconstructing the morality of the behavior, participants were asked to indicate which alternative best described the protagonist’s explanation after reading the vignette. Specifically, they were given two alternatives: “They state that it happened unintentionally and because something occurred for which they are not responsible” or “They state that they did it thinking that the action had no negative consequences”. The chi-square analysis conducted with the hits and misses in the four experimental conditions resulted in χ^2^(1) = 166.45 (*p* < 0.001), showing that most participants categorized each argument according to our expectation. However, to guarantee a comparative between both explanations, we excluded participants who did not correctly categorize these expressions (*N* = 32, 11.42%) for the analyses.

#### 2.1.4. Measured Variables

##### Manipulation Check

To ascertain that the vignette describing a positive or negative personal profile influenced the global attitude towards the targets, participants were asked to indicate the impression they had formed of the person featured in the vignette (1 = “very negative” to 7 = “very positive”). They were also asked to indicate the extent to which competence, morality, and sociability was a defining trait for the target (1 = “does not define them at all” to 7 = “defines them completely”).

##### Severity of the Behavior

While we selected behaviors of equal severity in this study, we also included the question “To what extent do you consider what this person has done to be severe?” (1 = “not severe at all” to 7 = “very severe”) in order to confirm that the information regarding the target of the behavior altered its perceived severity.

##### Dehumanization

We used the Infrahumanization Scale [32], which captures implicit tendencies and assesses the attribution of primary and secondary emotions. Participants were prompted to reflect on the target, and then they were asked the following: “To what extent do you think that person, in their daily life, may experience the following”? Using a 7-point scale ranging from 1 (not at all) to 7 (completely), participants indicated the perceived capacity of the targets to experience emotions. The scale included six primary emotions (excitement, joy, pleasure, fear, sadness, and anger) and six secondary emotions (friendship, compassion, hope, guilt, remorse, and shame). The internal consistency for the entire scale proved to be excellent in the current sample (α = 0.936), as well as in the subset focusing solely on the secondary emotions (α = 0.942). The emotional terms were selected based on a normative study [52], ensuring that they were effectively different in terms of humanness but had the same valence. Primary emotions exhibited lower levels of humanness (*M* = 2.55, *SD* = 0.47) compared to secondary emotions (*M* = 5.39, *SD* = 0.73; *t* (10) = 8.03, *p* < 0.001) on a scale ranging from 1 (shared by animals and humans) to 7 (exclusively human). However, both primary and secondary emotions showed similar levels of valence (*M* = 3.94, *SD* = 2.50 for primary; *M* = 3.44, *SD* = 2.10 for secondary; *t* (10) = 0.37, *p* = 0.716) on a scale from 1 (very unpleasant) to 7 (very pleasant).

#### 2.1.5. Procedure

At the beginning of the questionnaire, participants were informed of their rights and asked for their written consent. Each participant was randomly allocated to one experimental condition. They were first presented with one of the two descriptions of the target and asked to form an impression about them, indicating how likeable they found the target to be along with several questions about their personality. Then, participants read about an immoral action shared by that target in a previous study and one of the two moral rationalizations that the target had allegedly used when censured by others. Subsequently, the dependent measures of the study were introduced, followed by sociodemographic questions. Finally, participants were asked to indicate if their responses should be included for data analysis. To conclude the questionnaire, a debrief explained that the target was not a real person and that their profile had been created for experimental purposes to explore how perceptions are formed based on provided information.

#### 2.1.6. Statistical Analyses

We used the IBM SPSS Statistics 29 program for the analysis, with a significance level of 0.05. We carried out two 2 (profile of the target: positive vs. negative) × 2 (moral disengagement: reconstruction of agency vs. reconstruction of morality) analyses of variance (ANOVAs) to test our hypotheses about the main effects on the dependent variables (severity of the behavior, dehumanization). Additionally, we conducted a planned contrast analysis to confirm that a combination of the two independent variables (positive profile + agent reconstruction) led to a more favorable moral evaluation (perceived less severity of behavior and less dehumanization) compared to the other conditions.

### 2.2. Results

#### 2.2.1. Manipulation Check

Prior to analyzing the data, we sought assurance that our profile manipulation yielded the anticipated results. The analysis of responses regarding the valence of the target’s first impression showed that the participants who read the positive profile formed a significantly more favorable impression of the target (*M* = 6.58, *SD* = 0.73) compared to those who read the negative profile (*M* = 1.46, *SD* = 0.72; *t* (227) = 53.16, *p* < 0.001, *d* =−7.029, 95% CI [−7.72, −6.34]). Furthermore, after averaging the scores of sociability, competence, and morality (α = 0.95), the t-test also confirmed that the participants perceived the two profiles differently (*t* (227) = 25.49, *p* < 0.001, *d* = −3.37, 95% CI [−3.77, −2.97]) depending on whether they read the positive profile (*M* = 5.69, *SD* = 0.76) or the negative profile (*M* = 2.11, *SD* = 1.27). These preliminary tests ensured the effectiveness of our profile manipulation, setting the stage for the analysis of the collected data.

#### 2.2.2. Severity of the Behavior

**Figure 1:**
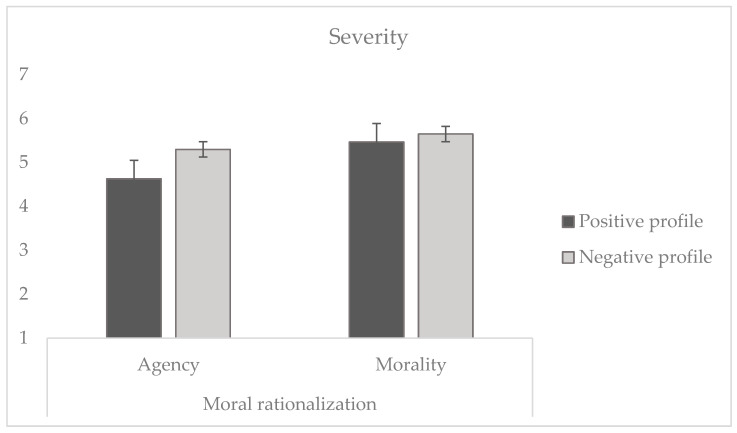
Severity attributed to immoral behaviors as a function of the profile of the target (positive vs. negative) and moral rationalization (reconstruction of agency vs. morality).

To test H1 (as transgressors are perceived as more likeable, the perceived severity of their actions decreases) and H2 (using reconstruction of agency rather than reconstruction of morality is associated with a further reduction in the perceived severity of the behavior), we conducted a 2 × 2 ANOVA. The results (see Figure 1) revealed a significant effect of the target’s profile (*F* (1, 225) = 5.25, *p* = 0.023, η_p_^2^ = 0.023), indicating that, in line with H1, participants considered the behavior as less severe when the profile was positive (*M* = 5.05, *SD* = 1.49) compared to when it was negative (*M* = 5.47, *SD* = 1.40).

Moreover, there was a significant effect of moral rationalization (*F* (1, 225) = 10.27, *p* = 0.002, η_p_^2^ = 0.044), showing that, in line with H2, participants considered the behavior of the target reconstructing agency as statistically less severe (*M* = 4.98, *SD* = 1.57) compared to the target reconstructing morality (*M* = 5.56, *SD* = 1.28). Finally, no significant interaction emerged between the profile of the target and moral rationalization (*F* (1, 225) = 1.74, *p* = 0.188). Additionally, to test the additive hypothesis H3, we conducted a planned contrast comparing the effect of the positive profile agent evading their responsibility against the one defending the benignity of the behavior or the negative profile. The result showed that, as expected, participants in this condition considered the transgression to be the least severe (*t* (225) = 50.39, *p* < 0.001).

#### 2.2.3. Dehumanization of the Transgressor

**Figure 2:**
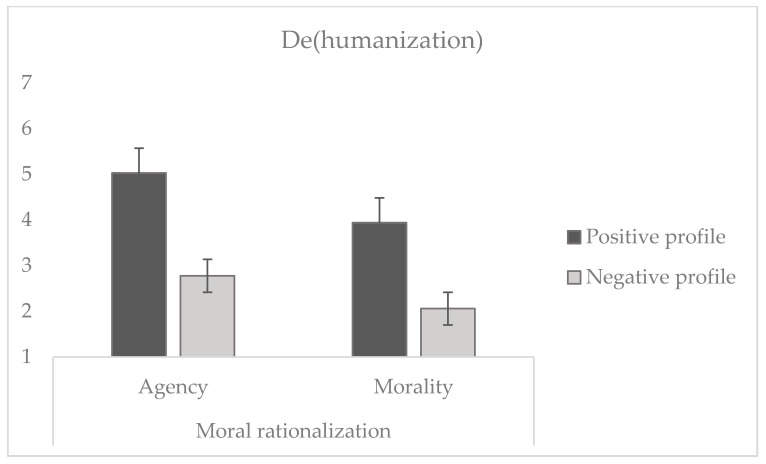
Dehumanization attributed to the transgressor of immoral behaviors as a function of the profile of the target (positive vs. negative) and moral rationalization (reconstruction of agency vs. morality). Note: lower scores indicate stronger dehumanization.

To test whether a transgressor is perceived as more human when their profile is positive rather than negative (H4) and when they use a reconstruction of agency explanation rather than reconstruction of morality (H5), we conducted a 2 × 2 ANOVA. The results (see Figure 2) revealed a significant effect of target profile (*F* (1, 225) = 174.66, *p* < 0.001, η_p_^2^ = 0.437), indicating that participants considered the positive profile (*M* = 4.49, *SD* = 1.34) as significantly more human compared to the negative profile (*M* = 2.45, *SD* = 1.17), in line with H4.

In addition, there was a significant effect of moral rationalization (*F* (1, 225) = 33.64, *p* < 0.001, η_p_^2^ = 0.130), since participants perceived that the target who reconstructed agency was more human (*M* = 3.84, *SD* = 1.75) compared to the target who reconstructed morality (*M* = 3.00, *SD* = 1.34), in line with H5. Lastly, there was no significant interaction between the profile of the target and moral rationalization (*F* (1, 225) = 1.41, *p* = 0.235). Then, to test the additive hypothesis that the least dehumanization would occur for the positive profile transgressor who justified their behavior by eluding responsibility (H6), we conducted a planned contrast of this condition against the three remaining ones (i.e., the positive profile agent defending the benignity of the behavior and both negative profile agents). This contrast was significant, *t* (225) = 37.09, *p* < 0.001, thus supporting H6.

### 2.3. Discussion

The results support our hypotheses, as both liking for the transgressor and rationalization of the behavior by reconstructing agency led participants to attribute less severity to the transgression and dehumanize the transgressor less. What is even more interesting is that the evaluations of both the behavior and the transgressor were more lenient when the agent was perceived as likeable and justified their behavior by eluding responsibility rather than by defending the benign nature of the action. However, is this result solely due to a relationship of liking based on personal characteristics, or can it be extrapolated to other relevant areas in interpersonal interaction? In Study 2, we operationalize likeability of the transgressor not through the description of a positive profile but by presenting them as ideologically similar to the participant.

## 3. Study 2

This study replicates the methodology of Study 1 but with a different manipulation of likeability towards the transgressor. Here, we presented a target with ideological similarity to the participant instead of describing an attractive personality profile. Research demonstrates that belief similarity is a robust source of interpersonal attraction [53,54,55]. Furthermore, recent studies show that moral judgments regarding transgressions are more tolerant in political contexts compared to personal ones [56]. Indeed, there are numerous everyday examples of how people judge the immoral behaviors of political leaders more leniently. A notable example involves former US president Donald Trump during his candidacy in 2016, making the unscrupulous remark that his supporters were so loyal that he could shoot someone on Fifth Avenue in New York and still not lose votes. This indicates that instead of having a notion of right and wrong independently of context, political ideology constitutes a source of instability in moral judgments, where attachment to political groups is a central component of identity [57,58,59]. In this regard, Walter and Redlawsk [60] found that people’s moral emotional responses depended on their identification with a political party. When a politician committed a moral violation, individuals who shared their ideology responded with less anger, contempt, disgust, and shame than those who did not share the same ideology. They also found that this effect was stronger among those who exhibited greater ideological identification with the political leader. Therefore, this study investigates whether an immoral action committed by a transgressor with whom there is ideological alignment affects the evaluation of the severity of the transgression and the dehumanization of the transgressor.

### 3.1. Methods

#### 3.1.1. Participants

British participants were recruited through an online research recruitment platform in exchange for a financial reward, and Qualtrics hosted the survey online. The G*Power analyses for the F tests (ANOVA: fixed effects, special, main effects, and interactions) with *p*-values α < 0.05, 95% power, and a medium effect size suggested that the target sample size should be 279 participants. Therefore, assuming a 15% error in the manipulation check response, we aimed to recruit 319 individuals. We asked the recruitment platform to distribute the study evenly to male and female participants with left-wing and right-wing political affiliations. Then, the data were screened to exclude participants who did not recognize the type of moral reasoning employed to justify the immoral action, failed the attention questions, or stated themselves not to analyze their answers. The final sample consisted of 246 participants with a mean age of 47.18 (*SD* = 15.03); 48.8% women, 50% men, and 1.2% other; and 50.2% left-wing.

#### 3.1.2. Design

To test our hypotheses, we conducted the same 2 (profile of the target: ideological matching vs. no ideological matching) × 2 (moral rationalization: reconstruction of agency vs. reconstruction of morality) between-subjects experimental design on the severity of the behavior and dehumanization of the transgressor variables as in Study 1.

#### 3.1.3. Material

##### Stimuli

(1) Likeability of the Target

We manipulated the level of likeability towards the target by varying their political beliefs. Participants were presented with the profile of a person who supposedly had participated in a previous study. The profile included information about their intelligence (stating that their score was slightly above average), decision-making (inclination towards long-term gains), personality (high sociability), social relationships (having a stable circle both personally and professionally), and views on various essential social issues (namely redistribution, abortion, LGBTQ+ rights, defense, and migration). In the latter variable, the person was presented as either a conservative or a progressive individual. The description of the conservative transgressor included phrases like “LM prefers promoting meritocracy because they believe personal success comes through individual effort”, while the progressive transgressor’s description included phrases like “LM prefers wealth redistribution with a focus on equal opportunities for all, regardless of their background or status”.

(2) Behaviors and Moral Rationalization

We utilized the same behaviors and explanations as those employed in Study 1. The chi-square analysis conducted with our expected correct and incorrect responses for moral rationalization strategies across the four experimental conditions yielded χ^2^(1) = 174.08 (*p* < 0.001), indicating that most participants categorized each argument according to our expectation. However, to guarantee a comparison between the two explanations, we again excluded participants who did not correctly categorize these expressions (*N* = 42, 13.16%).

#### 3.1.4. Measured Variables

We used the same measures as in Study 1 for dehumanization (Infrahumanization Scale) and severity of the behavior. Furthermore, we used two items to verify the effectiveness of the experimental manipulation. The first item aimed to assess ideological alignment with the transgressor: “To what extent does your ideology align with LM’s?” (1 = “not aligned at all” to 7 = “completely aligned”). The second item confirmed that ideological alignment led to a more positive impression of the transgressor: “Based on the information you have read about LM, what impression do you form of LM?” (1 = “very negative” to 7 = “very positive”). Additionally, in the sociodemographic variables, we queried their ideological position: “When discussing political ideology, terms like left and right are often used. On a scale of 1 to 10, where 1 means ‘more left’ and 10 ‘more right’, where would you place yourself”?

#### 3.1.5. Procedure

The experimental procedure was identical to Study 1.

#### 3.1.6. Statistical Analyses

To test our hypotheses, we followed the same analysis plan as in Study 1.

### 3.2. Results

#### 3.2.1. Manipulation Check

Prior to analyzing the data, we used the ideological matching measure to create the two groups. There were those who showed a high alignment with the transgressor’s ideology (*M* = 5.02, *SD* = 0.85, *N* = 127) and those who showed a low alignment (*M* = 1.97, *SD* = 0.78, *N* = 119, (*t* (244) = 29.33, *p* < 0.001, *d* = −3.73, 95% CI [−4.148, −3.32]). Importantly, there were no significant differences in ideological position between the high alignment group and the low alignment group (*t* (244) = 0.299, *p* = 0.766). However, as expected, participants in the high ideological alignment group formed a statistically significant more positive impression (*M* = 5.98, *SD* = 0.89) of the transgressor compared to those in the low alignment group (*M* = 4.26, *SD* = 1.59; (*t* (244) = 10.54, *p* < 0.001, *d* = −1.34, 95% CI [−1.618, −1.065]). These preliminary tests ensured the effectiveness of our profile manipulation, setting the stage for the analysis of the collected data.

#### 3.2.2. Severity of the Behavior

**Figure 3:**
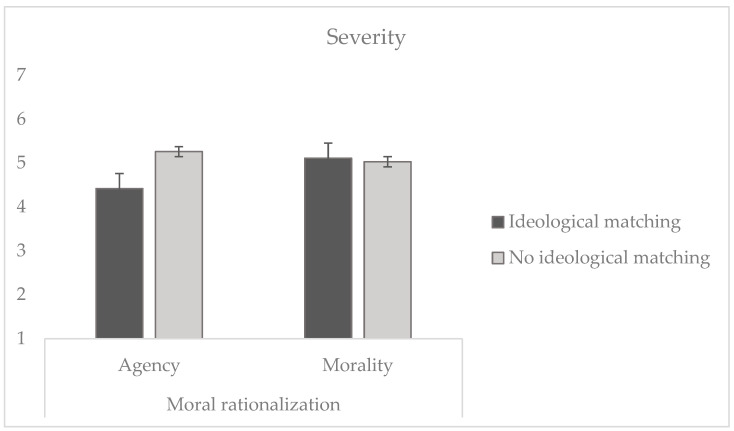
Severity attributed to immoral behaviors as a function of the profile of the target (ideological matching vs. no ideological matching) and moral rationalization (reconstruction of agency vs. morality).

The ANOVA tested whether ideological alignment with the transgressor led to a decreased perceived severity of their action (H1) and whether using reconstruction of agency rather than reconstruction of morality is associated with a further reduction in the perceived severity of the behavior (H2). The result yielded a statistically significant main effect of ideological alignment (*F* (1, 242) = 5.38, *p* = 0.021, η_p_^2^ = 0.022). In line with H1 (see Figure 3), participants considered the behavior of targets with ideological matching as less severe (*M* = 3.79, *SD* = 1.18) compared to the behavior of those with no ideological matching (*M* = 4.55, *SD* = 1.25). However, there was no significant effect of moral rationalization (*F* (1, 242) = 2.01, *p* = 0.158), showing that participants considered the behavior of the target who reconstructed agency to be as severe (*M* = 4.83, *SD* = 1.32) as the behavior of the one who reconstructed morality (*M* = 5.08, *SD* = 1.29); H2 is thus not supported. In this case, the analysis of the interaction showed a significant effect (*F* (1, 242) = 7.91, *p* = 0.005, η_p_^2^ = 0.032). Bonferroni post-hoc adjustment for pairwise comparisons indicated no significant difference (*p* = 0.333) in reconstructing agency and morality when the target had no ideological alignment.

However, this difference was significant when participants reported ideological alignment with the transgressor (*p* = 0.003). Still, to test H3, we conducted a planned contrast comparing the high alignment condition and reconstruction of agency against the other three conditions. The result of the analysis confirmed H3, as we obtained a statistically significant effect (*t* (242) = 3.872, *p* < 0.001).

#### 3.2.3. Dehumanization of the Transgressor

**Figure 4:**
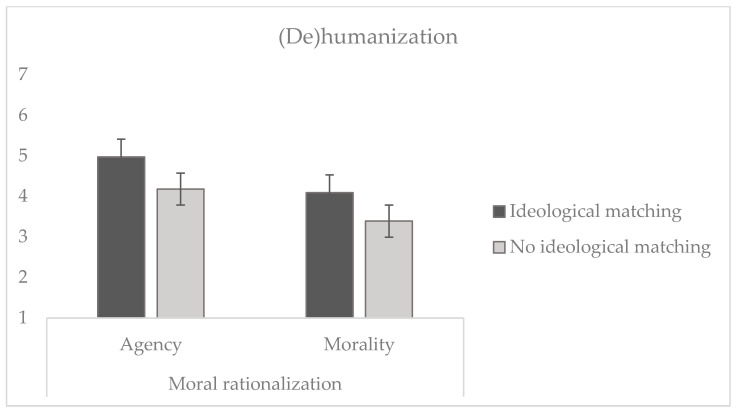
Dehumanization attributed to the transgressor of immoral behaviors as a function of the profile of the target (ideological matching vs. no ideological matching) and moral rationalization (reconstruction of agency vs. morality). Note: lower scores indicate stronger dehumanization.

To test whether participants dehumanize the transgressor less when there is ideological alignment with the transgressor (H4) or when they evade responsibility for their actions (H5), a 2 (high ideological alignment vs. low) × 2 (reconstruction of agency vs. reconstruction of morality) ANOVA was conducted. The analysis (see Figure 4) showed a significant effect of ideological alignment (*F* (1, 242) = 25.72, *p* < 0.001, η_p_^2^ = 0.096), indicating that, in line with H1, participants considered targets with ideological matching as more human (*M* = 4.55, *SD* = 1.25) compared to those with no ideological matching (*M* = 3.79, *SD* = 1.18). Additionally, in agreement with H5, there is a significant effect of moral rationalization (*F* (1, 242) = 32.30, *p* < 0.001, η_p_^2^ = 0.118). Specifically, participants considered the target who reconstructed agency to be more human (*M* = 4.59, *SD* = 1.24) compared to the target who reconstructed morality (*M* = 3.75, *SD* = 1.16).

Lastly, although there was no significant interaction between ideological alignment and moral rationalization (*F* (1, 242) = 0.093, *p* =0.761), the planned contrast between the high ideological matching condition and reconstruction of agency against the other three conditions confirmed H6, as a statistically significant effect was obtained (*t* (242) = 6.54, *p* < 0.001).

### 3.3. Discussion

Once again, higher levels of likeability towards the target resulted in lower assessment of the behavior’s severity and lower dehumanization of the transgressor. Similarly, when the target rationalized the behavior by reconstructing agency, there was less dehumanization compared to when they reconstructed morality. We also found that the positive impact of reconstructing agency instead of morality for evaluations of severity is only evident when there is a sense of likeability towards the target; there is no positive impact when there is no sense of likeability. It is important to note that Study 2 replicated the findings of likeability from Study 1 with a different manipulation of this variable. It is consistent with previous research where likeability has been manipulated in different ways, prompting benign moral evaluations [17]. Importantly, political alignment led to a more lenient moral judgment even when observers were judging immoral actions unrelated to the political realm.

## 4. General Discussion

In this research, we investigated whether attitudes towards a transgressor and the rationalization strategies they employ to justify their immoral action bias judgments of severity and attributions of humanness to the transgressor. The results demonstrate that likeability consistently worked to reduce the severity of the behavior and minimize the dehumanization of the perpetrator.

Therefore, our results underscore the effect achieved by the likeability evoked when describing someone with an admirable personality (Study 1) or someone with whom ideology is shared (Study 2). This finding demonstrates the potential for attitudes towards transgressors to bias moral judgments; the bias could result from attributing moral personality traits to the transgressor due to the correspondence bias and the halo effect generated by sympathy and liking [17,61]. In fact, it is plausible to assume that if moral traits are fundamental in our perceptions, any information about another person will impact the impression we form about their moral character to infer whether they are a reliable ally or a potential threat [49]. However, our results go beyond current research by specifying that the likeability bias not only modifies moral character attributions but also contributes to reducing the severity of the action and circumventing the tendency to dehumanize the transgressor. Moreover, consistent with recent research, receiving information that is not directly related to the behavior but that leads to a more favorable view of the target results in more lenient moral judgments [62]. This is an insightful finding because attitudes, which are omnipresent and unavoidable, can shape attributions of humanness even when engaging in immoral behavior [63]. The denial of full humanness to others serves as both the catalyst and outcome of conflicts, prejudice, and violence towards others, all too commonly witnessed in our daily lives [29,32]. Hence, it is important to discern the circumstances under which individuals are perceived as less human.

However, positive attitude towards the transgressor was not the only feature that proved to be decisive in the moral evaluation of the action and the transgressor. Moral rationalization, and especially a strategy based on agency reconstruction, proved to lead to more lenient moral judgments. In this study, we followed the recommendation of Schaefer and Bouwmeester [46] to separate the moral disengagement strategies proposed by Bandura et al. [43] from the specific mechanisms used to achieve them. These strategies are thus forms of rationalization whose logical processes invoke alternative moral codes or higher obligations aimed at selectively reconstructing reality to favor the transgressor. In this study, the process of reconstructing agency (“actor A is not responsible for behavior B”) was more effective than the process of reconstructing the morality of the action (“behavior B is not morally wrong”). Notably, this effect persisted even when the transgressor either outright denied committing the act or admitted to it but claimed a lack of intent. This result suggests that the tendency to cognitively restructure the agent’s role is a more efficient form of moral rationalization. Removing responsibility from the agent in an immoral action helped individuals dissociate from their internal moral standards and resolve potential cognitive dissonance associated with linking unethical behavior to someone they like [64,65]; it helps maintain a positive and socially acceptable self-image. Therefore, an important contribution of these studies is to show that not every strategy produces equally efficient results, a finding that is especially significant given the widespread use of these strategies and their varied consequences on social perception.

Lastly, this study anticipated that combining likeability towards the transgressor and the reconstruction of the transgressor’s agency would amplify the impact on the moral evaluation of both the action and the transgressor. The analysis of the planned contrasts confirmed this hypothesis. Consequently, this pioneering research demonstrates that a likeable transgressor who justifies their action can reduce the perceived severity of a transgression and avoid being dehumanized. Overall, our results shed light on the complexity of the process of moral judgments and offer some nuance to the likeability bias. Although we find confirmation of such bias, we also observe that not all rationalization strategies work to the same degree for those we like. Reconstruction of agency has a significatively better result when there is likeability towards the transgressor, whereas reconstructing morality has a similar effect regardless of liking. Reconstructing agency, then, has an especially positive effect for those we like, perhaps because our sympathy for them prompts us to readily detach them from the immoral action.

## 5. Limitations and Future Directions

We acknowledge that this research has several limitations that, at the same time, offer valuable insights for future research. First, we manipulated likeability using profiles based on all three dimensions of the Stereotype Content Model (competence, warmth, and morality). Future studies could examine whether focusing on only one or two dimensions yields similar results and even compare their effects. Additionally, exploring the use of the agency and communion framework could provide further insights into how likeability functions when focusing on different aspects of social perception. Second, despite having two different manipulations of likeability, both are text-based. Future research could explore different approaches to manipulating liking using other sources, such as the perceived attractiveness of individuals’ faces [61]. Likeability was also manipulated in both cases via the personal characteristics of the targets, but future research could include considerations of inter-group belongingness [66]. Furthermore, a more complex but exciting possibility would be to combine these approaches—for instance, examining the impact of personally liking a target from an out-group vs. disliking another target from the in-group.

There is a limitation in our manipulations of rationalization strategies, as the agency reconstruction ones are not fully conceptually identical across the two behaviors. In the rumor scenario, the transgressor shifts agency by claiming that someone started the rumor, which, if believed, could influence participants’ judgment. This is precisely the intent of the rationalization strategy. However, this approach is less applicable to the intercourse scenario, where the transgressor does not deny the immoral act, making it unlikely that participants fully absolve them of responsibility. Future research could examine the credibility of each strategy, as this may impact their effectiveness. Still, this variability may also enhance the ecological validity of our study, reflecting the diverse interpretations of moral justifications in real-life contexts and their impact on perceptions.

Another caveat of this study is that each transgressor utilized only one rationalization strategy. However, given the rapidity and complexity of social interactions, individuals are likely to employ multiple explanations simultaneously. Hence, it becomes imperative to consider a range of rationalization methods and their potential interactions to avoid arriving at ambiguous and incomplete conclusions. We argue that another interesting avenue for future research would be studying whether different strategies are more effective depending on the participant’s political ideology, as each ideology tends to prioritize certain moral values. In addition, examining the distinction between the intentionality a person claims after the misbehavior and the actual intent they had before engaging in it could provide deeper insights into how perceived intentionality influences moral judgments [67] and the efficacy of different rationalization strategies. While this study represents an initial exploration of the distinct impact of a single strategy on dehumanization, further research is necessary to comprehensively understand the interplay of multiple rationalization methods and their implications for perceptions of humanness.

A final limitation would be that we used two transgressive behaviors in each of the studies. Therefore, caution should be exercised when generalizing the conclusions of this research. Future investigations could explore the impact of liking or the moral rationalization strategies on different types of behaviors—for instance, analyzing whether the transgression itself is moralized to a greater or lesser extent.

## 6. Implications

People tend to believe that their moral judgments are as objective as scientific statements [15,16]. This belief leads them to categorically label people and behaviors as good or bad based on whether they transgress prevailing moral norms [68]. It even drives them to take more or less extreme actions to restore the subverted moral code. However, our findings indicate that this perceived objectivity is not true to reality, as we find that both likeability and the use of rationalization strategies can bias these evaluations. This discrepancy between perceived objectivity and actual judgement has important implications. First, it may affect interpersonal relations because people consistently disfavor those whom they initially dislike, generating an entrenchment of such a negative evaluation. This would validate the popular wisdom behind sentences like “You never get a second chance to make a first impression”. This dynamic could easily translate to workplace environments where potential immoral actions have crucial consequences and for which evaluation may also be biased. The factors we investigated could sway the judgment of a person in charge, favoring those they like when they commit reprehensible actions. This could ultimately undermine the procedural justice of the whole organization, undermining legitimacy, creating discontent, and worsening performance [69]. In addition, crucially, in many justice systems, bystanders provide information about the facts or even decide what type of punishments or rehabilitation to provide by acting as jurors, witnesses, or other social agents. Moreover, our findings could also shed light on how media narratives shape moral judgments of public figures, such as politicians, by influencing audience evaluations based on their likeability. Additionally, politicians may also be biased towards certain groups or individuals due to their own likeability, which could impact their stance on policy issues. Therefore, the insights gained from this research can inform the development of interventions aimed at educating jurors, legal professionals, organizations’ HR and managers, journalists, politicians, and the general public about the influence of bystander biases on evaluating the morality of others’ actions.

Regarding the process of rationalization more specifically, our results suggest that reconstruction of agency is a superior mechanism to escape negative judgments compared to reconstruction of morality. This also has important implications in many of the processes described, as those acting as judges, whether it is in legal processes or in more everyday contexts, should be especially attentive towards those being judged using this strategy. Being aware that we are more prone to favor those who reconstruct their agency could improve our capacity to judge those who do so.

## 7. Conclusions

Moral judgment, rather than being an objective assessment, is influenced by attitudinal variables such as the likeability evoked by the transgressor in the observer or the explanations used to rationalize the transgression. Recognizing the impact of these variables is crucial for fostering a deeper understanding of moral decision-making processes in interpersonal contexts with moral implications.

## Data Availability

The materials and original data presented in the study are openly available in osf at https://osf.io/mdxcy/?view_only=3a0c1d71b14a4adca8ff07b5a3802da5, accessed on 12 December 2024.
